# Deletion of TRPV4 enhances in vitro wound healing of murine esophageal keratinocytes

**DOI:** 10.1038/s41598-020-68269-8

**Published:** 2020-07-09

**Authors:** Ammar Boudaka, Claire T. Saito, Makoto Tominaga

**Affiliations:** 1 Department of Physiology, College of Medicine and Health Sciences, Sultan Qaboos University, Al-Khoud, P.O. Box 35, 123 Muscat, Sultanate of Oman; 2 Division of Cell Signaling, National Institute for Physiological Sciences, Okazaki, Aichi, 444-8787 Japan; 3 Department of Physiological Sciences, SOKENDAI (The Graduate University for Advanced Studies), Okazaki, Aichi, 444-8787 Japan; 4 Thermal Biology Group, Exploratory Research Center on Life and Living Systems, Okazaki, Aichi, 444-8787 Japan

**Keywords:** Physiology, Cell biology, Cell migration

## Abstract

Transient receptor potential vanilloid 4 (TRPV4) is a non-selective cation channel that is widely expressed in different body tissues and plays several physiological roles. This channel is highly expressed in esophageal keratinocytes where its activation mediates ATP release. However, whether TRPV4 has a role in wound healing of esophageal keratinocytes is unclear. In this study, we demonstrated that both cell migration and proliferation were slower in wild-type esophageal keratinocytes compared to cells having TRPV4 knockout. Our results suggest that TRPV4-mediated release of ATP from esophageal keratinocytes contributes to a decrease in the rate of in vitro wound healing via the ATP degradation product adenosine, which acts on A_2B_ adenosine receptors.

## Introduction

The esophagus is a muscular tube that provides a conduit for food from the pharynx to the stomach, and is critical for swallowing of food. Sentient responses to mechanical, thermal and chemical stimuli also occur in the esophagus. The esophageal mucosa has a highly specialized epithelium that forms an important protective barrier against diverse chemical and physical insults^1^. The esophageal epithelium comprises non-keratinized stratified squamous epithelium that is susceptible to injury due to the continuous exposure to various stimuli ranging from a swallowed food bolus to gastric refluxate at the distal end of the esophagus that results from an incompetent lower esophageal sphincter in gastroesophageal reflux disease^1^. The abovementioned insults could cause esophageal erosions and in more severe cases can lead to ulceration. Several factors affect epithelial wound healing including heat and Ca^2+^ ions^2,3^. Calcium regulates the growth, differentiation and apoptosis of many cell types, including epidermal keratinocytes, in which Ca^2+^ induces G1/G0 cell cycle arrest^4^. However, the exact molecular mechanisms that regulate wound healing of esophageal mucosa are still largely unknown.

TRP channels are non-selective cation channels that mediate influx of Ca^2+^, Mg^2+^ and monovalent cations into different cell types^5^. A functional TRP channel has a central hydrophilic channel pore surrounded by four subunits, which each consist of six transmembrane segments with cytoplasmic C and N termini^6,7^. To date, mammalian TRP channels are a large superfamily comprising 28 members that are categorized into six subfamilies based on the their amino acid arrangement: ankyrin (TRPA1), canonical (TRPC1-7), melastin (TRPM1-8), mucolipin (TRPML1-3), poly-cystin (PC) (TRPP1-3), and vanilloid (TRPV1-6)^5,8,9^. These channels are generally expressed in various body tissues and many cells express one or more subtypes. TRP channels regulate several different physiological cellular processes, such as cell proliferation, apoptosis, cell death, mechanosensation, cell volume regulation, secretion, control of vascular permeability and blood vessel tone as well as angiogenesis^10–14^. Moreover, TRP channel activation can be achieved by a large array of physical and chemical stimuli including mechanical forces, heat, cold, ions and small molecules^15,16^. Thus, TRP channels are crucial players in multiple facets of health and disease^17^.

TRPV4 was initially reported to be an osmo- or mechano-sensor^18,19^ that can be activated by moderate warmth (> 27 °C)^20,21^, UV light^22^ and endogenous substances such as arachidonic acid and its cytochrome P450-derived metabolites (epoxyeicosatrienoic acids), endocannabinoids (anandamide and 2-arachidonoylglycerol)^23^, as well as by diverse exogenous chemical stimuli including GSK1016790A, the synthetic phorbol ester 4α-phorbol 12,13-didecanoate (4α-PDD)^21,24,25^. The antagonist HC-067047 inhibits TRPV4 activity^26^. TRPV4 is widely expressed throughout the body including hippocampal neurons^27^, endothelial cells^21,28^, esophageal^13^, gastric^14^ and urinary bladder epithelia^26^ as well as skin keratinocytes^29^, where it contributes to numerous physiological processes. We previously showed that TRPV4 is highly expressed in the mucosa lining the esophagus in mice and mediates ATP release from cultured esophageal keratinocytes in response to mechanical, chemical and thermal stimuli^13,30^. Based on its activity as a thermo- and mechano-sensor, and the roles played by heat and calcium in wound healing, TRPV4 is a possible candidate modulator that could have significant physiological effects in wound healing.

In this study, we investigated the putative role of TRPV4 in esophageal keratinocytes migration and proliferation. We decided to use the cell insert assay instead of a traditional wound healing assay with scratching in order to evaluate the covered gap area more precisely. Our results indicate that TRPV4-mediated ATP release from esophageal keratinocytes slows in vitro wound healing via a degradation product of ATP, adenosine, which acts on A_2B_ adenosine receptors.

## Results

### Effects of TRPV4 deletion on gap closure of esophageal keratinocytes

**Figure 1:**
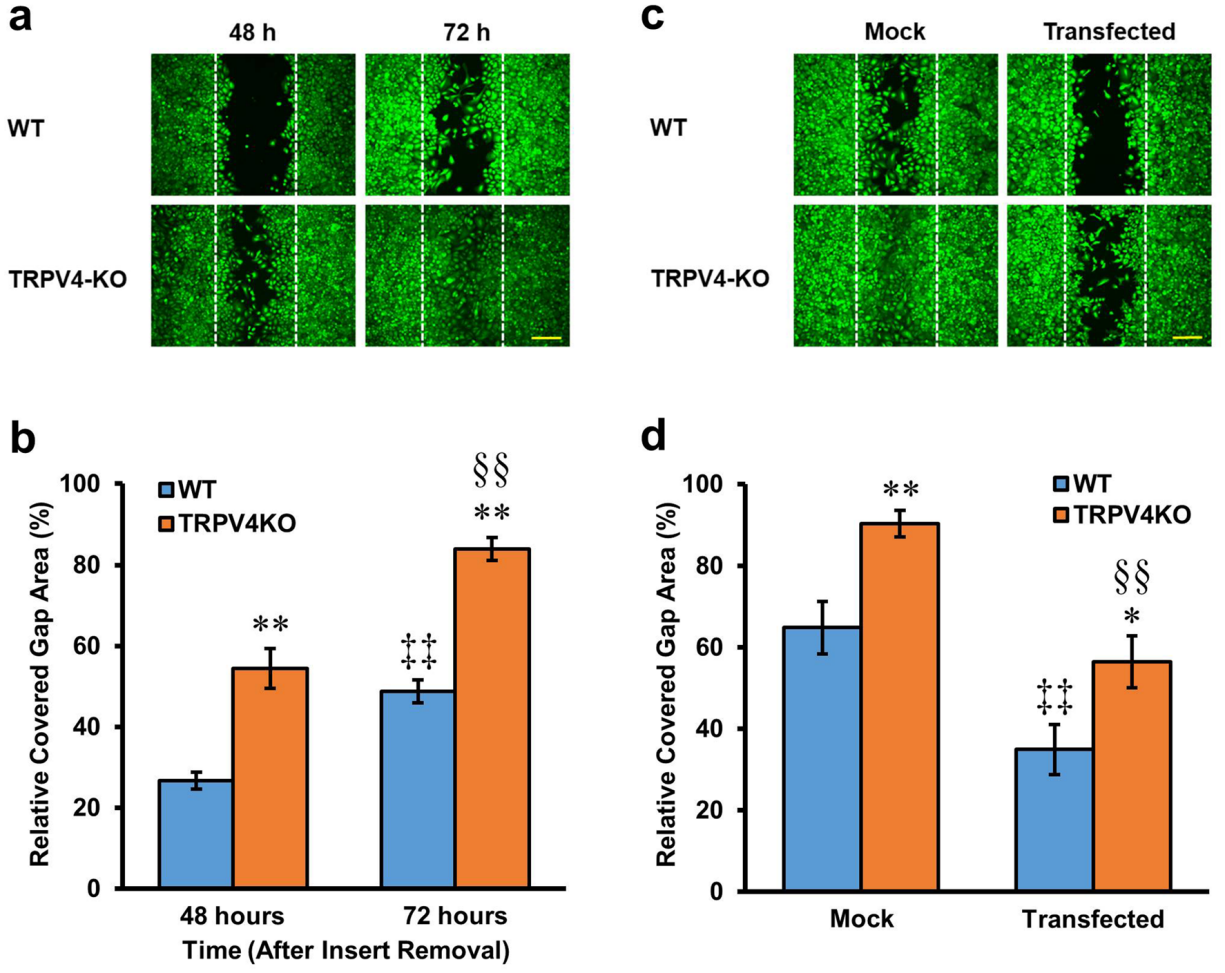
The effect of TRPV4 deletion on gap closure of esophageal keratinocytes. (**a**) Representative images of cultured WT and TRPV4-KO keratinocytes stained with calcein. (**b**) The percentage of covered gap area was significantly larger 48 and 72 h after insert removal for cultured TRPV4-KO keratinocytes compared to WT. Data are presented as means ± SEM (n = 18–24). **p < 0.01 compared with WT, ^‡‡^*p* < 0.01 compared with WT 48 h, ^§§^*p* < 0.01 compared with TRPV4-KO 48 h; as determined by t-test. (**c**) Representative images of cultured WT and TRPV4-KO keratinocytes transfected with mouse TRPV4 cDNA and stained with calcein. (**d**) Rescue transfection of TRPV4-KO esophageal keratinocytes with mouse TRPV4 cDNA significantly delayed gap closure. WT keratinocytes transfected with TRPV4 also exhibited significantly delayed gap closure. Data are presented as means ± SEM (n = 6–9). *p < 0.05; **p < 0.01 compared with WT, ^‡‡^*p* < 0.01 compared with mock-transfected WT, ^§§^*p* < 0.01 compared with mock-transfected TRPV4-KO; as determined by t-test. White dotted lines indicate the edges of the cell-free gap at 0 h post-insert removal. Scale bars represent 200 μm.

To gain insights into the pathophysiological role of TRPV4 in cell migration and proliferation, we performed comparative in vitro wound healing studies^31^ using TRPV4-KO and WT control mice. We first assessed the cellular purity of cultured esophageal keratinocytes using CK14 immunoreactivity, and confirmed that nearly all of the cultured keratinocytes obtained from both strains were CK14-immunoreactive (Supplementary Fig. S1). Time-lapse experiments showed that TRPV4-KO keratinocytes had enhanced migration compared to WT cells (Supplementary Video S1). Moreover, the wound healing assay revealed that the percentages of covered gap area were significantly larger for TRPV4-KO compared to WT esophageal keratinocytes at both 48 and 72 h after insert removal (48 h: 54.5 ± 4.9% vs. 26.7 ± 2.1%; 72 h: 84.0 ± 2.8% vs. 48.8 ± 2.8%; n = 18–24; *p* < 0.01) (Fig. 1a,b). Furthermore, for both mouse strains, the percentages of covered gap area after insert removal were significantly larger at 72 h than at 48 h (WT: 48.8 ± 2.8% vs. 26.7 ± 2.1%; TRPV4-KO: 84.0 ± 2.8% vs. 54.5 ± 4.9%; n = 18–24; *p* < 0.01) (Fig. 1b). Based on these results, we performed follow up assessments of gap closure at 72 h after removal of the insert unless stated otherwise.

Next, we examined how transfecting esophageal keratinocytes with mouse TRPV4 cDNA affected gap closure. Transfected cells of both strains were identified by their red fluorescence under the microscope (Supplementary Fig. S2). For both WT and TRPV4-KO cultures, the transfection significantly reduced the percentage of covered gap area compared to the respective mock-transfected controls (WT: 34.9 ± 6.2% vs. 64.8 ± 6.5%; TRPV4-KO: 56.4 ± 6.4% vs. 90.3 ± 3.3%; n = 6; *p* < 0.01) (Fig. 1c,d). However, the gap closure of transfected WT keratinocytes was significantly slower than transfected TRPV4-KO cells (34.9 ± 6.2% vs. 56.4 ± 6.4%; n = 6; *p* < 0.05) (Fig. 1d).

### Effects of TRPV4 deletion on the cell cycle of esophageal keratinocytes

**Figure 2:**
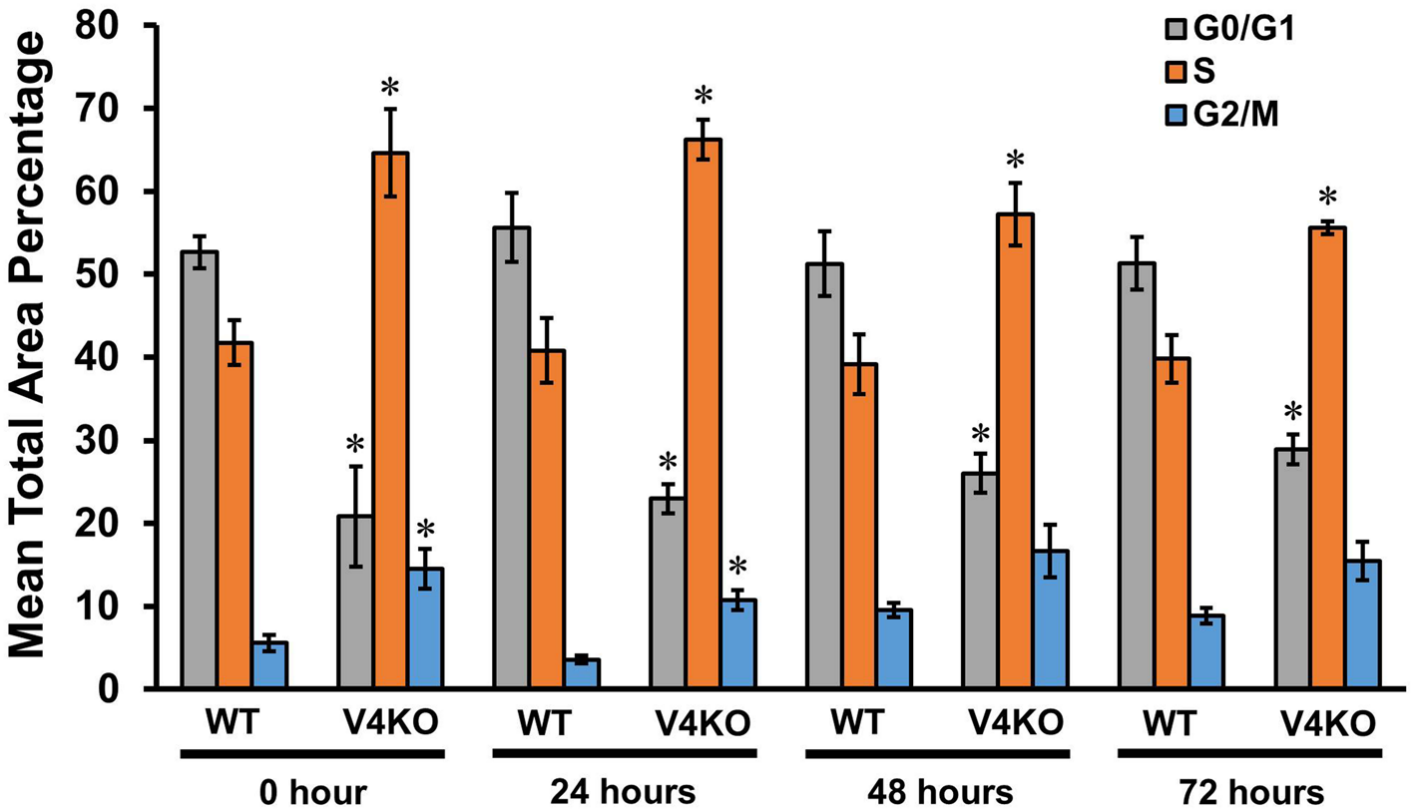
The effect of TRPV4 deletion on different phases of the cell cycle in esophageal keratinocytes. The percentages of cells in the G0/G1 (gray bars), S (orange bars) and G2/M phase (blue bars) in WT and TRPV-KO (V4KO) cultures at 0, 24, 48 and 72 h after insert removal. Data are presented as means ± SEM (n = 6–9). *p < 0.05 compared with the respective phase in WT cultures as determined by t-test.

Since TRPV4 deficiency was associated with enhanced gap closure, we next checked whether TRPV4 had any effect on esophageal keratinocyte proliferation. A cell cycle assay revealed that the percentage of cells in the G0/G1 phase was significantly smaller among TRPV4-KO esophageal keratinocytes compared to the respective WT cultures at 0, 24, 48 and 72 h after insert removal (20.9 ± 6.1% vs. 52.7 ± 1.9% at 0 h; 23.0 ± 1.8% vs. 55.6 ± 4.2% at 24 h; 26.1 ± 2.4% vs. 51.3 ± 3.9% at 48 h; 28.9 ± 1.8% vs. 51.3 ± 3.2% at 72 h; n = 6–9; *p* < 0.05) (Fig. 2). On the other hand, at all four time points after insert removal, the percentage of cells in the S phase was significantly larger among TRPV4-KO esophageal keratinocytes compared to the respective WT cultures (64.6 ± 5.3% vs. 41.8 ± 2.7% at 0 h; 66.3 ± 2.4% vs. 40.8 ± 3.9% at 24 h; 57.3 ± 3.8% vs. 39.2 ± 3.6% at 48 h; 55.6 ± 0.8% vs. 39.8 ± 2.9% at 72 h; n = 6–9; *p* < 0.05). Significantly more TRPV4-KO cells were in the G2/M phase compared to WT cultures at 0 and 24 h after insert removal (14.5 ± 2.4% vs. 5.6 ± 1.0% at 0 h; 10.8 ± 1.2% vs. 3.6 ± 0.5% at 24 h; n = 6–9; *p* < 0.05), but at 48 h and 72 h after removal, the percentages were similar (16.7 ± 3.2% vs. 9.6 ± 0.9% at 48 h; 15.5 ± 2.3% vs. 8.9 ± 0.9% at 72 h; n = 6–7; *p* = 0.178 and 0.082, respectively) (Fig. 2 and Supplementary Fig. S3). These results suggest that TRPV4 has a modulatory role in the proliferation of esophageal keratinocytes which could partly contribute to the difference in gap closure.

### Effects of cyclic tensile strain on gap closure

**Figure 3:**
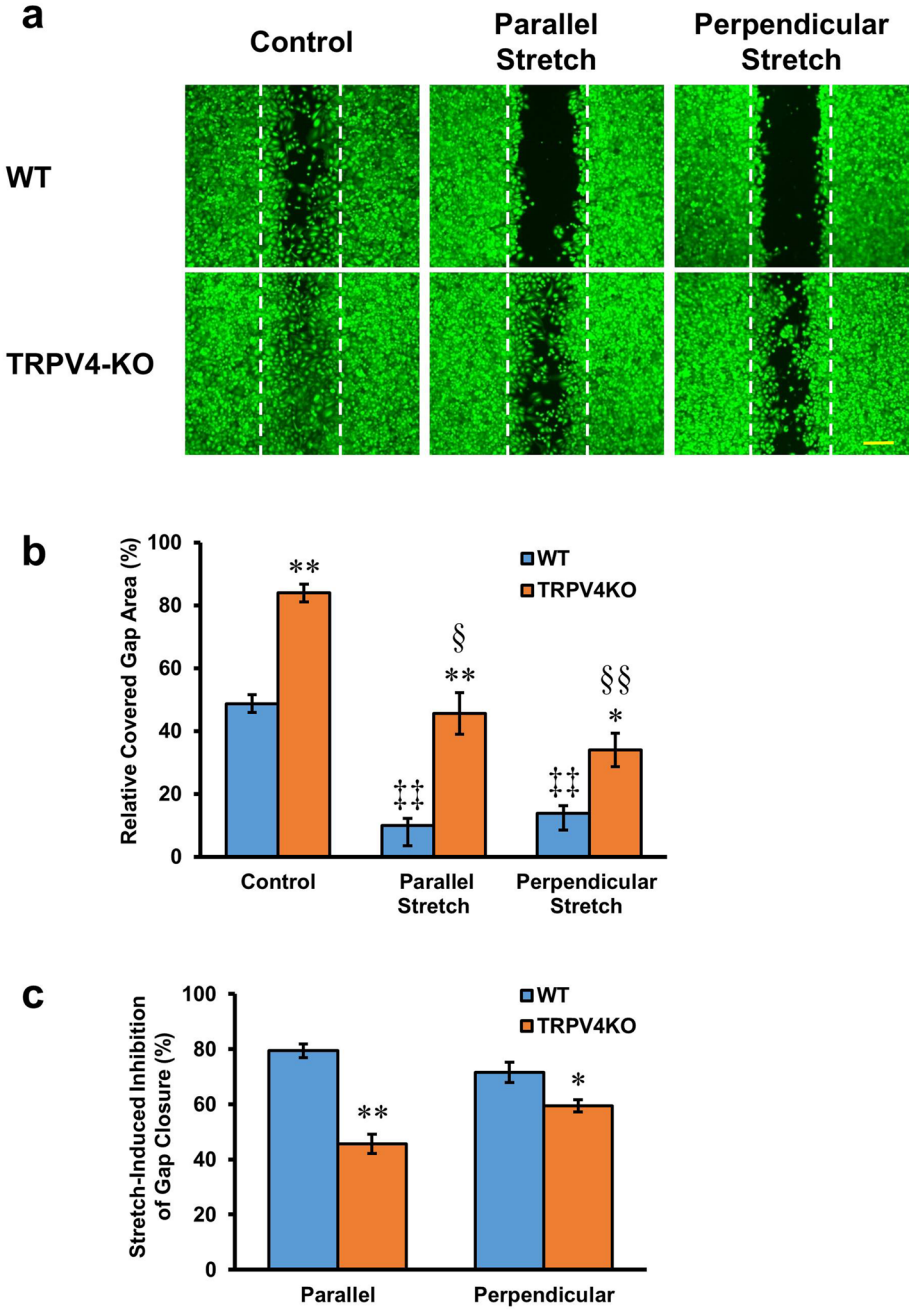
Cyclic tensile strains delay gap closure of esophageal keratinocytes. (**a**) Representative images of cultured WT and TRPV4-KO keratinocytes exposed to 20% stretch at a frequency of 0.33 Hz for 72 h before calcein staining. The stretch was applied parallel or perpendicular to the gap alignment. White dotted lines indicate the edges of cell-free gap at 0 h post-insert removal. Scale bar represents 200 μm. (**b**) The percentage of covered gap area in WT and TRPV4-KO cultures following parallel or perpendicular stretch for 72 h. (**c**) Percent inhibition of gap closure induced by parallel or perpendicular stretch in WT and TRPV4-KO keratinocyte cultures. Data are presented as means ± SEM (n = 10–12). *p < 0.05; **p < 0.01 compared with WT, ^‡‡^*p* < 0.01 compared with WT control, ^§§^*p* < 0.05; ^§§^*p* < 0.01 compared with TRPV4-KO control; as determined by t-test.

Cells sense the mechanical properties of their surrounding environment and activate intracellular signaling pathways that play important roles in cell survival, proliferation, differentiation and migration. Since the TRPV4 channel is a mechanoreceptor, we assessed the effect of stretch on in vitro wound healing of esophageal keratinocytes. After insert removal, we exposed the cultured keratinocytes to cyclic tensile strains applied either perpendicular or parallel to the direction of the cell-free gap, as the direction of cyclic stretching can affect gap closure^32^. Cyclic tensile strains, applied either perpendicular or parallel to the cell-free gap, significantly decreased the percentage of covered gap area in WT cultured esophageal keratinocytes compared to cultures not exposed to mechanical stress (10.1 ± 2.2% and 13.8 ± 2.4% for parallel and perpendicular stretch, respectively, vs. 48.8 ± 2.9% for the control; n = 10–12; *p* < 0.01) (Fig. 3a,b). Cyclic tensile strains also significantly reduced the percentage of covered gap area in TRPV4-KO cultured esophageal keratinocytes, although to a lesser extent than for WT (45.6 ± 6.6% and 34.1 ± 5.3% for parallel and perpendicular stretch, respectively, vs. 84.0 ± 2.8% for the control; n = 10–12; *p* < 0.05 and < 0.01, respectively) (Fig. 3a,b). Similarly, in esophageal keratinocyte cultures from WT and TRPV4-KO mice exposed to cyclic tensile strain, the percentage of covered gap area for WT was significantly smaller than that for TRPV4-KO (10.1 ± 2.2% vs. 45.6 ± 6.6% for parallel stretch and 13.8 ± 2.4% vs. 34.1 ± 5.3% for perpendicular stretch; n = 10–12; *p* < 0.01 and < 0.05, respectively) (Fig. 3b). The percentages of covered gap area were similar after exposure to parallel or perpendicular stretch (WT: 10.1 ± 2.2% for parallel stretch vs. 13.8 ± 2.4% for perpendicular stretch; TRPV4-KO: 45.6 ± 6.6% for parallel stretch vs. 34.1 ± 5.3% for perpendicular stretch; n = 10–12; *p* = 0.104 and 0.155, respectively). Moreover, the percent inhibition of gap closure by cyclic tensile strains, both parallel and perpendicular, was significantly larger in WT compared to TRPV4-KO cells (79.4 ± 2.5% vs. 45.7 ± 3.6% for parallel stretch and 71.6 ± 3.7% vs. 59.4 ± 2.2% for perpendicular stretch; n = 10–12; *p* < 0.01 and < 0.05, respectively) (Fig. 3c).

### Effects of exocytotic ATP release on gap closure

**Figure 4:**
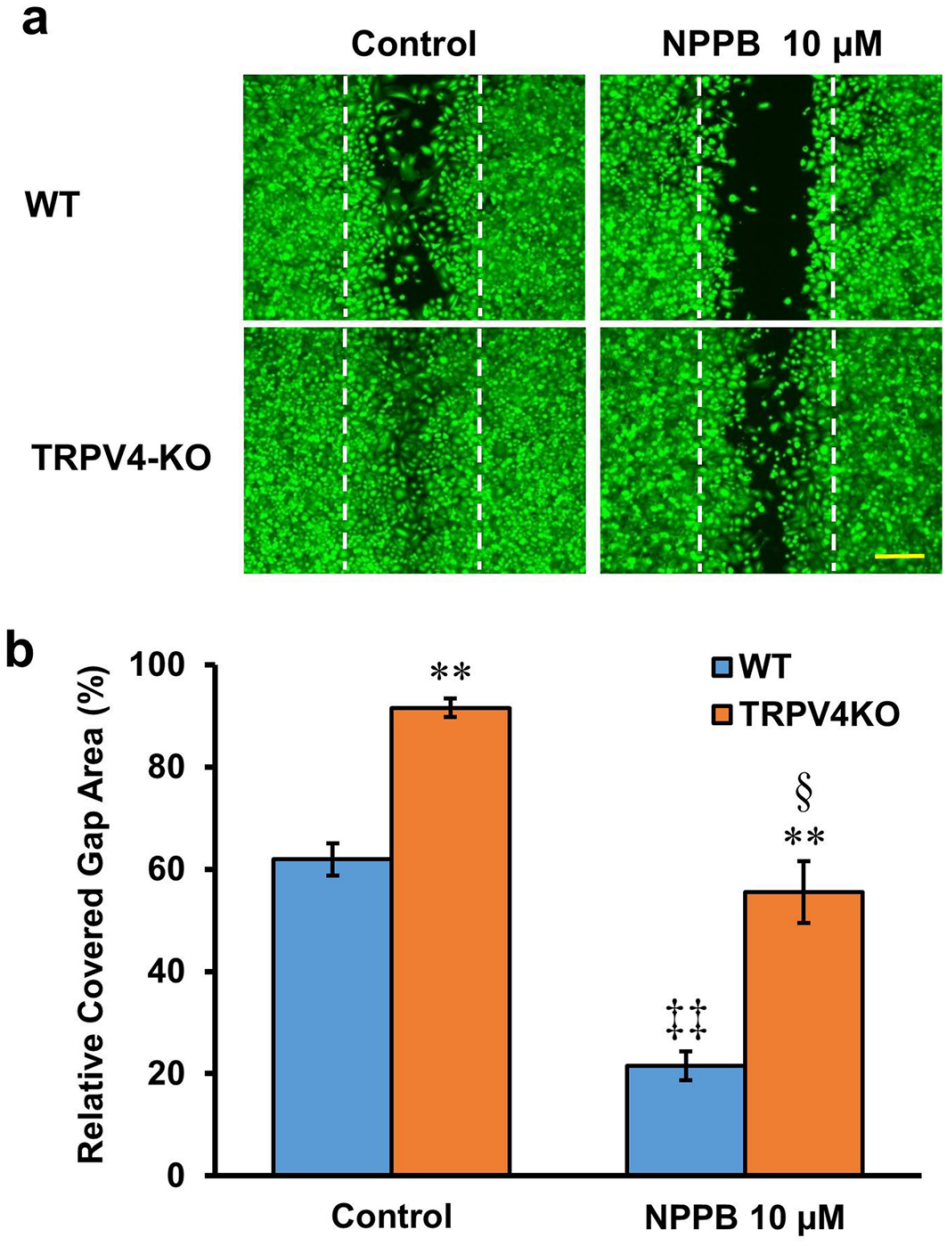
Stimulation of exocytotic ATP release delays gap closure. (**a**) Representative images of WT and TRPV4-KO keratinocytes treated with 10 µM NPPB before staining with calcein. White dotted lines indicate the edges of the cell-free gap at 0 h after insert removal. The scale bar represents 200 μm. (**b**) The effect of 10 µM NPPB on percentage of covered gap area in WT and TRPV4-KO keratinocyte cultures. Data are presented as means ± SEM (n = 6–9). **p < 0.01 compared with WT, ^‡‡^*p* < 0.01 compared with WT control, ^§^*p* < 0.05 compared with TRPV4-KO control; as determined by t-test.

We previously showed that esophageal keratinocytes release ATP in response to TRPV4 stimulation^13^. Therefore, we next investigated whether the exocytotic release of ATP from keratinocytes can affect gap closure. WT and TRPV4-KO keratinocytes treated with 10 µM NPPB, a stimulant of cellular ATP release through exocytosis^13,33^, showed significantly reduced percentages of covered gap area compared to their respective controls (21.5 ± 2.8% vs. 61.9 ± 3.2% for WT and 55.6 ± 6.1% vs. 91.6 ± 1.8% for TRPV4KO; n = 6–9; *p* < 0.01 and < 0.05, respectively) (Fig. 4a, b). The percentages of covered gap area for cultured mouse keratinocytes treated with 10 µM NPPB were significantly larger for TRPV4-KO keratinocytes compared to WT cells (55.6 ± 6.1% vs. 21.5 ± 2.8%; n = 6; *p* < 0.01) (Fig. 4b).

### Effects of exogenous ATP and ATP hydrolase on gap closure

**Figure 5:**
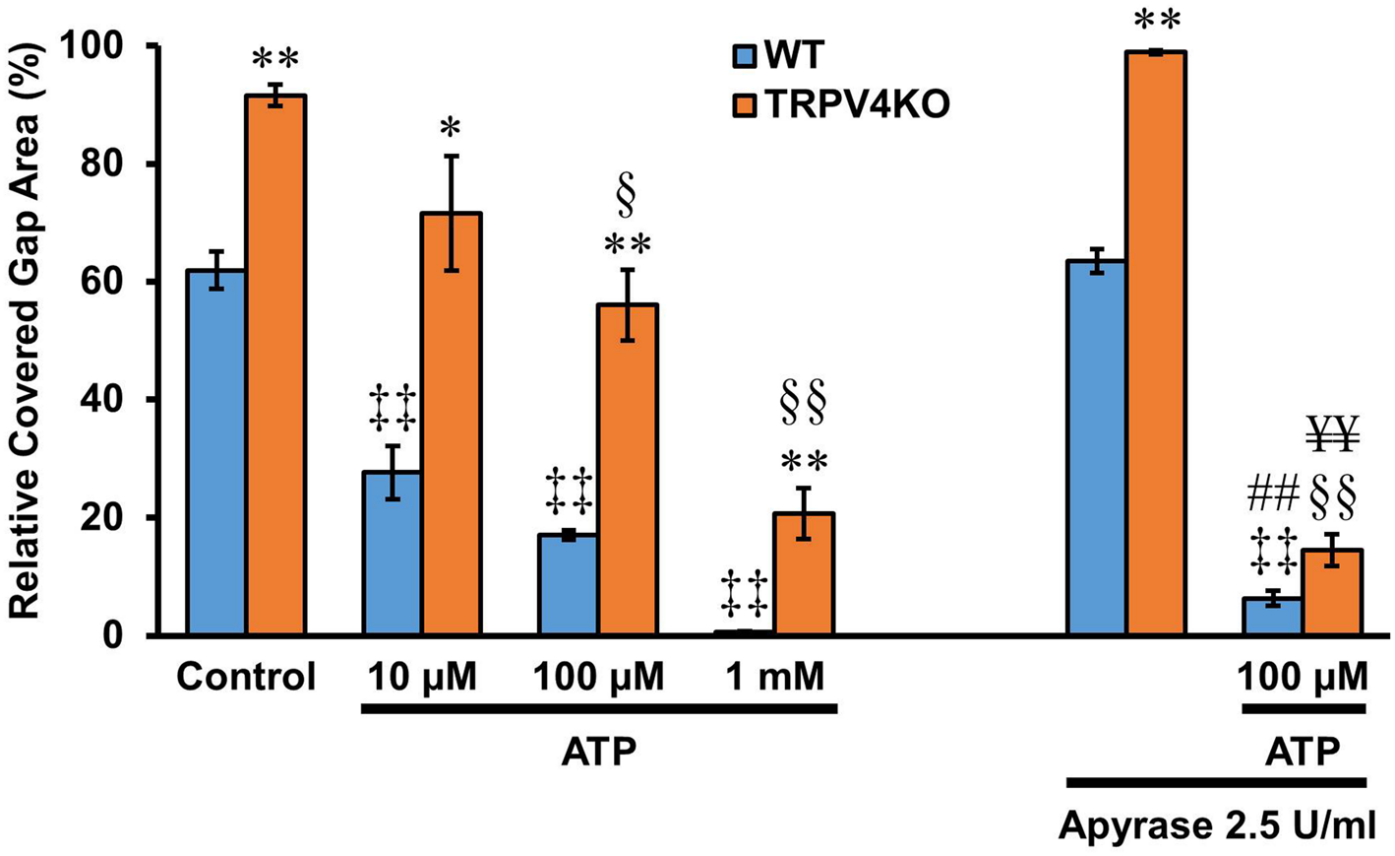
Exogenous ATP inhibits esophageal keratinocyte gap closure. The effect of ATP (10–1,000 µM) on the percentage of covered gap area in WT and TRPV4-KO keratinocyte cultures is shown on the left. On the right, the effect of 2.5 U/ml apyrase (ATP hydrolase) on the inhibition of gap closure mediated by 100 µM ATP is shown. Data are presented as means ± SEM (n = 6–9). *p < 0.05; **p < 0.01 compared with WT, ^‡‡^*p* < 0.01 compared with WT control, ^§^*p* < 0.05; ^§§^*p* < 0.05 *p* < 0.01 compared with TRPV4-KO control, , ^##^*p* < 0.01 compared with WT 100 μM ATP, ^¥¥^*p* < 0.01 compared with TRPV4-KO 100 μM ATP; as determined by one-way ANOVA followed by Tukey post-hoc test.

We also tested the effect of exogenous ATP on in vitro wound healing. ATP (10–1,000 µM) treatment was associated with a significant, concentration-dependent reduction in the percentages of covered gap area for both WT and TRPV4-KO cell cultures compared to their respective controls (WT: 27.7 ± 4.5%, 17.1 ± 0.8% and 0.6 ± 0.1% for 10 µM, 100 µM and 1 mM ATP, respectively, vs. 61.9 ± 3.2% for control; TRPV4KO: 71.6 ± 9.7%, 56.0 ± 6.0% and 20.8 ± 4.3% for 10 µM, 100 µM and 1 mM ATP, respectively, vs. 91.6 ± 1.8% for control; n = 6–9; *p* < 0.01–0.05) (Fig. 5, left). Moreover, the inhibitory effect of exogenous ATP on gap closure was significantly larger for WT relative to TRPV4-KO cells at all ATP concentrations tested (27.7 ± 4.5% vs. 71.6 ± 9.7% for 10 µM ATP, 17.1 ± 0.8% vs. 56.0 ± 6.0% for 100 µM ATP and 0.6 ± 0.1% vs. 20.8 ± 4.3% for 1 mM ATP; n = 6–9; *p* < 0.01–0.05) (Fig. 5, left).

To check whether ATP itself or one of its degradation products is responsible for the inhibitory effects on gap closure, the ATP hydrolase apyrase was added to the culture medium. Apyrase (2.5 U/ml) did not affect the percentage of covered gap area in either WT or TRPV4-KO keratinocyte cultures compared to their respective controls (WT: 63.4 ± 2.0% vs. 61.9 ± 3.2%; TRPV4KO: 98.9 ± 0.4% vs. 91.6 ± 1.8%; n = 6–9; *p* = 0.09 and 0.69, respectively; Fig. 5, right). However, apyrase (2.5 U/ml) did markedly potentiate the inhibitory effect of exogenous ATP (100 µM) on gap closure in both WT and TRPV4-KO cell cultures compared to their respective controls (WT: 6.4 ± 1.3% vs. 61.9 ± 3.2%; TRPV4KO: 14.5 ± 2.7% vs. 91.6 ± 1.8%; n = 6–9; *p* < 0.01) (Fig. 5, right). Moreover, apyrase (2.5 U/ml) significantly potentiated the inhibitory effect of exogenous ATP (100 μM) on gap closure in both WT and TRPV4-KO cell cultures compared to their respective responses to exogenous ATP (100 μM) in the absence of apyrase (WT: 6.4 ± 1.3% vs. 17.1 ± 0.8%; TRPV4-KO: 14.5 ± 2.7% vs. 56.0 ± 6.0%; n = 6–9; *p* < 0.01) (Fig. 5).

### Effects of exogenous adenosine on gap closure

**Figure 6:**
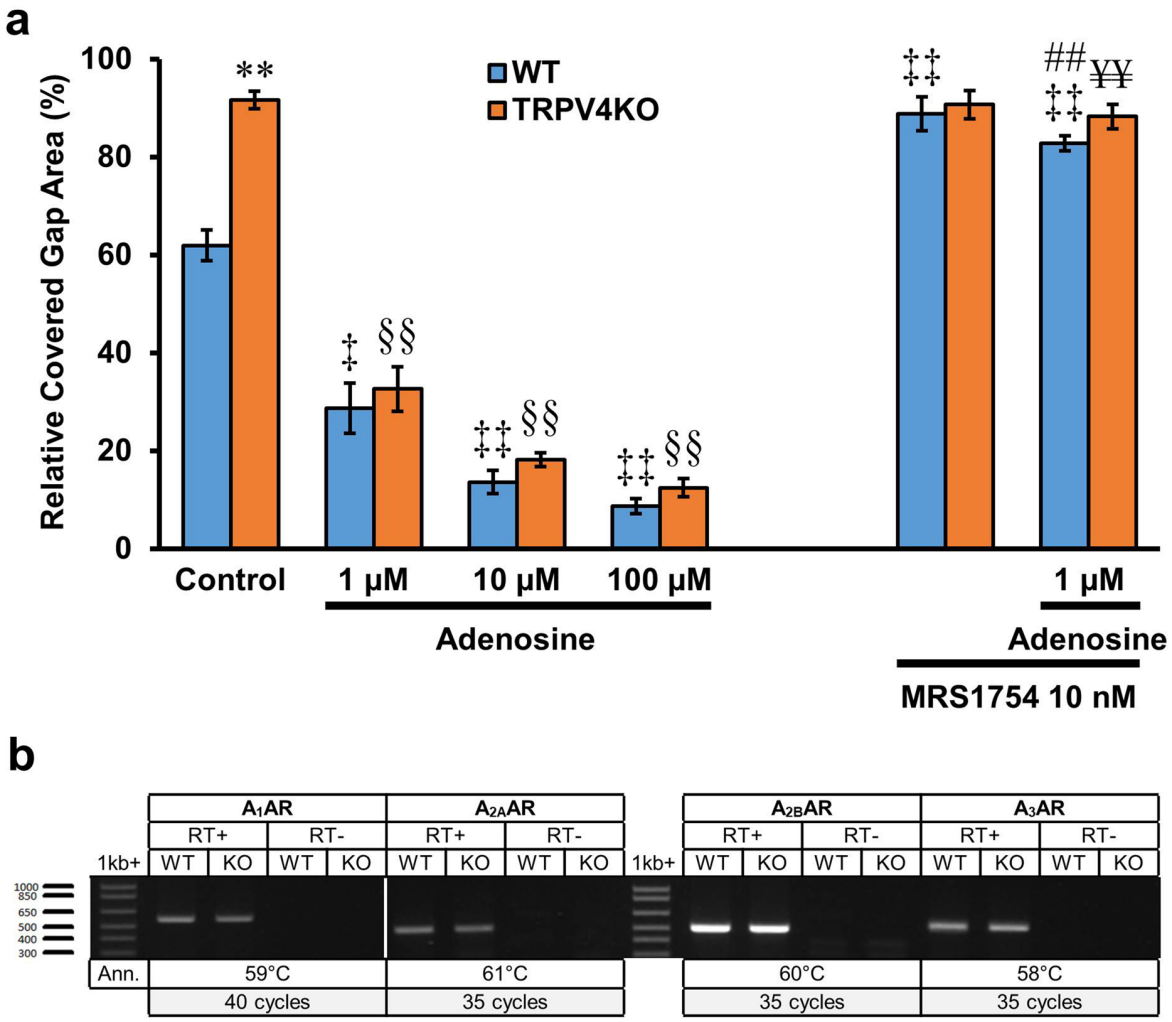
Adenosine inhibition of gap closure can be reversed by treatment with an adenosine receptor antagonist. (**a**) Exogenous adenosine inhibits esophageal keratinocyte wound healing. The effect of adenosine (1–100 µM) on the percentage of covered gap area in WT and TRPV4-KO keratinocyte cultures was examined. Cells were also treated with 10 nM MRS1754 (selective A_2B_ adenosine receptor antagonist) to examine its effect on in vitro wound healing inhibition mediated by 1 µM adenosine. Data are presented as means ± SEM (n = 6–12). *p < 0.05; **p < 0.01 compared with WT, ^‡^*p* < 0.05; ^‡‡^*p* < 0.01 compared with WT control, ^§§^*p* < 0.01 compared with TRPV4-KO control, ^##^*p* < 0.01 compared with WT 1 μM adenosine, ^¥¥^*p* < 0.01 compared with TRPV4-KO 1 μM adenosine; as determined by one-way ANOVA followed by Tukey post-hoc test. (**b**) Adenosine receptor mRNA transcription was examined with ( +) and without (–) RT reaction. All adenosine receptor subtypes were transcribed in the esophageal mucosa of WT and TRPV4-KO mice, but A_2B_ adenosine receptor had apparently higher transcription compared to A_1_, A_2A_ and A_3_ adenosine receptors. Full-length gels are shown in Supplementary Fig. S7.

Since ATP hydrolysis by apyrase significantly increased the inhibitory effect of exogenous ATP on in vitro wound healing of WT and TRPV4-KO keratinocytes, we next examined whether the ATP degradation product adenosine can indirectly mediate the observed inhibitory effect of ATP. Exogenously applied adenosine (1–100 µM) significantly and concentration-dependently reduced the percentages of covered gap area in both WT and TRPV4-KO cell cultures compared to their respective controls (WT: 28.7 ± 5.1%, 13.6 ± 2.3% and 8.7 ± 1.6% for 1 µM, 10 µM and 100 µM adenosine, respectively, vs. 61.9 ± 3.2% for control; TRPV4KO: 32.6 ± 4.5%, 18.2 ± 1.4 and 12.4 ± 1.9% for 1 µM, 10 µM and 100 µM adenosine, respectively, vs. 91.6 ± 1.8% for control; n = 6–12; *p* < 0.01–0.05) (Fig. 6a, left). The response of WT and TRPV4-KO keratinocytes to the inhibitory effect of exogenous adenosine at all adenosine concentrations tested was similar (28.7 ± 5.1% vs. 32.6 ± 4.5% for 1 µM adenosine, 13.6 ± 2.3% vs. 18.2 ± 1.4% for 10 µM adenosine and 8.7 ± 1.6% vs. 12.4 ± 1.9% for 100 µM adenosine; n = 6–12; *p* = 0.148, 0.109 and 0.066, respectively) (Fig. 6a, left).

### mRNA transcription of adenosine receptors in murine esophageal mucosa

Four subtypes of adenosine receptors have been identified: A_1_AR, A_2A_AR, A_2B_AR, andA_3_AR^34,35^. To investigate which subtype(s) mediate the inhibitory effect of exogenous adenosine, we assessed the expression pattern of different adenosine receptor subtypes in esophageal mucosa from WT and TRPV4-KO mice. Esophageal mucosa from both WT and TRPV4-KO mice showed mRNA expression of all four adenosine receptor subtypes, with apparently higher transcription levels of A_2B_ receptor (Fig. 6b). Moreover, *Ck14* (keratinocyte marker), *Slc17a9* (a gene encoding vesicular nucleotide transporter) and *Gapdh* were transcribed in the mucosa of both strains (Supplementary Fig. S4). As expected, TRPV4 mRNA transcription was only detected in the mucosa of WT and not in that of TRPV4-KO mice (Supplementary Fig. S4S4).

### Effects of selective A_2B_ adenosine receptor blocker on gap closure

Given the observation of apparently higher expression levels of A_2B_ adenosine receptor in esophageal mucosa from both mouse strains, we checked the effect of the selective A_2B_ adenosine receptor antagonist, MRS1754, on gap closure. Treatment of WT cultures with MRS1754 (10 nM) significantly increased the percentages of covered gap area to levels that were comparable to that of TRPV4-KO cultures (88.8 ± 3.4% vs. 90.7 ± 2.9%; n = 6; *p* = 0.69) (Fig. 6a, right). Moreover, MRS1754 (10 nM) significantly reversed the inhibitory effect of exogenous adenosine (1 μM) on gap closure in both WT and TRPV4-KO cell cultures relative to the response seen for cells treated with exogenous adenosine (1 μM) alone (WT: 82.8 ± 1.6% vs. 28.7 ± 5.1%; TRPV4-KO: 88.3 ± 2.5% vs. 32.6 ± 4.5%; n = 6; *p* < 0.01) (Fig. 6a).

## Discussion

Wound healing is an activity of utmost importance for living organisms. Even under culture conditions, most cellular layers exhibit the capacity to restore injured areas via migration and proliferation. Due to their location and characteristic functions, epithelial tissues are frequently used as models to study basic aspects of healing processes both in vitro and in vivo. Cell migration to an injury site is crucial for repair after injury. In this study, we showed that deletion of TRPV4 enhances migration of esophageal keratinocytes and that transfection of TRPV4 cDNA into esophageal keratinocytes harvested from TRPV4-KO mice slowed in vitro wound healing. A similar effect was observed in WT esophageal keratinocytes transfected with TRPV4 cDNA; this result could be because TRPV4 overexpression following transfection of these WT cells could further suppress in vitro wound healing compared with mock-transfected WT control cells. Therefore, our findings suggest that TRPV4 has a modulatory role on esophageal keratinocytes, where it can affect cell migration and proliferation.

TRPV4 is a non-selective cation channel that has high permeability to calcium^5^. A previous study showed the ability of calcium to induce cell cycle arrest in skin keratinocytes^4^. Our cell cycle assay revealed that TRPV4 deletion markedly increased the fraction of esophageal keratinocytes in the S and G2/M phases, which further supports our observation of enhanced gap closure in TRPV4-KO keratinocytes compared to WT cells during a 72 h period after insert removal. In WT cultures, the fraction of keratinocytes in the G2/M phase increased to levels comparable to the fraction seen for TRPV4-KO cultures at 48 and 72 h after insert removal, which could be attributed to a delayed onset of increases in the WT cell proliferation rate compared to their TRPV4-KO counterparts. The observed arrest of WT keratinocytes in the G0/G1 phase of the cell cycle suggests that, in addition to its modulatory effect on cell migration, TRPV4 has a regulatory role in keratinocyte proliferation.

TRPV4 channels function as mechanoreceptors^36^. However, to the best of our knowledge, the effect of mechanical stress on esophageal keratinocyte migration was unclear. The results of the current study clearly showed that cyclic cell stretch markedly inhibited in vitro wound healing in WT esophageal keratinocytes compared to TRPV4-KO cells. The inhibition of gap closure that was observed, albeit to a lesser extent, for esophageal keratinocytes isolated from TRPV4-KO mice could be due to the presence of other mechanoreceptors expressed by these keratinocytes. These other mechanoreceptors might, in turn, have a modulatory role on cell migration and proliferation. The direction of stretch did not significantly affect gap closure in either WT or TRPV4-KO cultures, which is inconsistent with findings from a previous study that showed a directional dependence of cyclic stretch-induced cell migration in wound healing of *Xenopus laevis* epithelial-like cell monolayers^32^. These varying results could be attributed to species-specific differences.

Numerous studies have indicated the ability of many cell types to release ATP^13,37^ in response to multiple stimuli including subjecting cell membranes to stretch^12,13^. The released ATP plays various physiological roles including inhibition of cell proliferation^38,39^, which is consistent with our observation in the current study that exogenous ATP inhibited gap closure. A similar effect was observed with stimulation of exocytotic ATP release using NPPB. Although NPPB is widely used as an inhibitor of many chloride channels^40–42^, it was shown to stimulate vesicular exocytosis from cultures esophageal keratinocytes and other secretory epithelial cell lines^13,33^. However, its inhibitory effect on cell migration via blockage of chloride channel^40^ cannot be ruled out in our study and needs further future investigation.

We have previously shown that TRPV4 stimulation mediates exocytotic ATP release from esophageal keratinocytes and that constitutively larger amounts of ATP are released from WT esophageal keratinocytes compared to TRPV4-KO cells^13^, which could explain our observation of a stronger inhibitory effect of exogenous ATP on gap closure in WT cells as TRPV4 contributes to the amount of constitutively released ATP. Although we proposed that ATP release in response to TRPV4 stimulation could be responsible for the slower gap closure seen for WT keratinocyte cultures, the inability of apyrase to affect gap closure or negate the inhibitory effect of exogenous ATP rules out a direct role for ATP in modulating in vitro wound healing.

Ectonucleotidases are extracellular enzymes that degrade extracellular ATP to yield different products including adenosine^43–45^. Adenosine is a naturally occurring nucleoside that controls several physiological processes, including cell proliferation via the activation of G-protein-coupled adenosine receptors (AR)^35,44^. Therefore, we hypothesized that adenosine, as an ATP degradation product, could be a candidate molecule involved in modulating in vitro wound healing of esophageal keratinocytes. Our results clearly demonstrated the ability of exogenous adenosine to markedly and concentration-dependently inhibit gap closure in both WT and TRPV4-KO cultures. This finding is supported by results from a previous study involving analysis of purine compounds that were present in the culture medium during cell exposure to ATP. This previous study revealed that more than 95% of the added ATP was metabolized within 1 h and that there was an increase in accumulation of purine metabolites, including adenosine, at higher concentrations of added ATP^38^. Taking into account the ability of WT keratinocytes to constitutively release larger amounts of ATP^13^ compared to TRPV4-KO cells, we expected to see higher levels of extracellular ATP catabolites, suggesting that these compounds may act via adenosine receptors to regulate cell replication. Four subtypes of AR have been identified: A_1_AR, A_2A_AR, A_2B_AR, and A_3_AR^34,35^. A_2A_AR and A_2B_AR subtypes are reported to exert contrasting effects on skin keratinocyte proliferation, which is stimulated by A_2A_AR and inhibited by A_2B_AR^46^. These results are consistent with our findings, wherein a selective A_2B_AR antagonist markedly enhanced gap closure in WT keratinocytes to levels that were comparable to TRPV4-KO cells, and significantly blocked the inhibitory effect of exogenous adenosine on gap closure in cultures of cells isolated from both mouse strains. At the physiological nanomolar range, adenosine mainly activates A_1_AR, A_2A_AR, and A_3_AR, whereas A_2B_AR activation requires micromolar concentrations^47^, which further supports the possibility of A_2B_AR involvement in our experiment since we used adenosine at a concentration of 1–100 μM. Moreover, the ability of A_2B_AR antagonist to enhance gap closure in WT keratinocytes, which were not treated with exogenous adenosine, further supports our hypothesis that adenosine, as a degradation product of constitutively released ATP, could be a candidate molecule involved in modulating in vitro wound healing of esophageal keratinocytes.

In WT cultures, the constitutively released ATP is likely metabolized by ectonucleotidases, expressed by keratinocytes, to ADP, AMP and ultimately to adenosine^38,48^, which could explain the lack of any additional effect for exogenous apyrase on gap closure of WT keratinocytes in the absence of exogenous ATP. Additionally, the degradation of exogenous ATP into ADP, AMP and adenosine, that add to the constitutively release ATP and its metabolites, could explain the higher ability for exogenous ATP to inhibit gap closure in WT compared to TRPV4KO cultures. This difference in inhibition of gap closure between WT and TRPV4KO keratinocytes disappeared when apyrase accelerated the degradation of exogenous ATP to its ultimate metabolite adenosine, an effect close to the effect of exogenous adenosine.

Our RT-PCR results showed that the A_2B_ receptor subtype had apparently high transcription level among the different subtypes in the esophageal mucosa, whereas genes encoding the A_1_, A_2A_ and A_3_ receptors were only weakly transcribed. These data are in accordance with previous observations for other cell types, including murine skin keratinocytes, in which A_2B_AR is reported to be the only adenosine receptor that is expressed to significant levels^46,49–52^. To our knowledge, this is the first description of the expression pattern of the adenosine receptors in murine esophageal mucosa. Our results suggest a predominant role for A_2B_ AR subtype in the physiological control of in vitro wound healing of esophageal keratinocytes.

In conclusion, in the present study we have shown for the first time that TRPV4 delays esophageal keratinocyte in vitro wound healing through its contribution to increased levels of adenosine, derived from TRPV4-mediated ATP release, that act via A_2B_AR. Our results suggest that TRPV4 and adenosine could be promising novel therapeutic targets for esophageal erosions and ulcers.

## Materials and methods

### Animals

Male wild-type (WT) (C57BL/6NCr) mice (8–14 weeks old; SLC) were used as a control. TRPV4-deficient (TRPV4-KO) mice^53^ were backcrossed on a C57BL/6NCr background. Mice were housed in a controlled environment (12 h light-12 h dark cycle; room temperature, 22–24 °C; 50–60% relative humidity) with free access to food and water. All procedures involving the care and use of animals were approved by The Institutional Animal Care and Use Committee of the National Institutes of Natural Sciences and carried out in accordance with the guidelines of the National Institute for Physiological Sciences.

### Chemicals

MCDB 153, ATP, adenosine, NPPB, apyrase and MRS1754 were purchased from Sigma-Aldrich (St. Louis, MO, USA).

### Primary culture of esophageal keratinocytes

Mice of both strains were killed by cervical dislocation following anesthesia with pentobarbital. The entire length of the esophagus was dissected and placed in cold (4 °C) PBS lacking Ca^2+^ and Mg^2+^. The muscle layer was removed using forceps and the remaining inner tissue containing the keratinous layer was incubated in 1 ml 0.25% trypsin solution (Invitrogen) at 4 °C for 8 h. The gelatinized submucosal layer and lamina propria mucosae were gently peeled away and the keratinocytes were harvested with a cell scraper (Greiner Bio-one). The obtained cells were filtered with a cell strainer (BD Falcon) and resuspended to a density of 1 × 10^6^ cells/ml and seeded in 2-well culture inserts placed on glass bottom dishes (Matsunami, Tokyo, Japan). The cells were incubated in MCDB 153 medium^13^ containing 5 μg/ml insulin, 0.4 μg/ml hydrocortisone, 14.1 μg/ml phosphorylethanolamine, 10 ng/ml epidermal growth factor (all from Sigma), 10 μg/ml transferrin (Funakoshi, Japan), 40 μg/ml bovine pituitary gland extract (Kyokuto, Japan), 25 μg/ml gentamicin, 50 U/ml penicillin, 50 μg/ml streptomycin and 10% fetal bovine serum (FBS). After incubating cells at 37 °C in 5% CO_2_ for 24 h, the medium was changed to a medium lacking FBS. Medium with freshly added agonist or antagonist was changed every day as dictated by the experimental design.

### Wound healing assay

The cell insert assay was used to evaluate in vitro wound healing instead of a traditional wound healing assay with scratching in order to determine the covered gap area more precisely. Esophageal keratinocytes were seeded in 2-well culture inserts (ibidi, Germany) on glass bottom dishes (Matsunami, Tokyo, Japan) and incubated for 72 h to reach confluence. The culture inserts were then gently removed, leaving a well-defined 500-µm cell-free gap. The regularity of the cell-free gap was checked by phase-contrast microscopy immediately after insert removal and only those cultures that had regular gap edges were used for further experiments (Supplementary Fig. S5). Gap closure was followed for 24, 48 or 72 h. At the end of each experiment the cells were washed with FBS-free MCDB 153 medium and loaded with calcein acetoxymethyl ester (Dojindo, Kumamoto, Japan) (1:1,000 in FBS-free medium) for 15 min at 37 °C to allow cell visualization. Images were obtained using a fluorescence microscope (BZ-9000; Keyence Corporation, Osaka, Japan) and analyzed with ImageJ software (National Institutes of Health, Bethesda, MD, USA) (Supplementary Fig. S6). To avoid any possible long-term toxic effect of calcein on cultured cells, live cell staining with calcein was performed only at the end of each experiment. For time-lapse analysis, a 35 mm culture dish containing cells that were cultured in 2-well inserts after insert removal was placed on a stage top incubator (Tokai Hit Glass Heating Stage, Japan) of a confocal laser scanning microscope (IX83 Olympus, Japan). Images were captured every 15 min for 72 h using cellSens imaging software (Olympus, Tokyo, Japan). Then time-lapse videos were created (Supplementary Video S1).

### Transfection

Esophageal keratinocytes were seeded, as described above, in 2-well culture inserts and left for 72 h to reach confluence. The keratinocytes were then transfected with mouse TRPV4 cDNA with a C-terminal linked DsRed cDNA using Lipofectamine (Invitrogen) and the transfected cells were incubated for an additional 24 h prior to insert removal and culture medium change. Mock-transfected cultures of both strains were used as controls. Gap closure of transfected esophageal keratinocytes was followed for 72 h before the cells were loaded and visualized with calcein. Images were analyzed as described above.

### Cell cycle assay

Esophageal keratinocytes were seeded as described for the wound healing assay until the cells reached confluence. The culture insert was then removed and the cells were stained either immediately (0 h) or after 24, 48 or 72 h with a Cell-Clock Cell Cycle Assay Kit (Biocolor, United Kingdom). Images were taken using a BZ-9000 microscope (Keyence Corporation, Osaka, Japan) and analyzed with ImageJ software. The distribution of cell cycle phases was defined as the threshold color and expressed as percentages. G2/M phase (dark blue) cells were defined as hue 0–255, saturation 40–255, and brightness 0–90. S phase (green) cells were defined as hue 70–255, saturation 40–255, brightness 90–255. G0/G1 phase (yellow green) cells were defined as hue 0–70, saturation 40–255, and brightness 90–255.

### Application of cyclic tensile strain to keratinocyte monolayer cultures

Esophageal keratinocytes were seeded in 2-well culture inserts placed inside stretch chambers having a culture surface area of 2 cm × 2 cm (Menicon Life Science, Nagoya, Japan) and allowed to reach confluence over 72 h. After insert removal, mechanical stress was applied using a ShellPa mechanical cell strain instrument (Menicon Life Science, Nagoya, Japan). The chamber was attached to a stretching apparatus that was pre-installed in the culture incubator. This apparatus had one fixed side opposite a movable side that can be driven by a computer-controlled motor. Using this apparatus, the entire silicon membrane area and almost all the cells on the stretch chambers can be stretched uniformly^54,55^. In the current study, a cyclic tensile strain (0.33 Hz, 20% elongation) was applied for 72 h. Cells without mechanical stress were seeded on the same chambers and used as controls. At the end of the experiment, cells were loaded and visualized with calcein as described above. The percentage of stretch-induced inhibition of gap closure was then calculated using the non-stretched control cultures as a reference.

### RT-PCR

Total mRNA was purified from esophageal mucosa using Sepasol-RNA I Super G (Nacalai Tesque). Reverse transcription was performed using Super Script III reverse transcriptase (Invitrogen) for 40 min at 50 °C. To investigate mRNA transcription of different adenosine receptor subtypes (A_1_AR, A_2A_AR, A_2B_AR, A_3_AR), CK14, TRPV4 and VNUT, DNA fragments were amplified using EmeraldAmp PCR Master Mix (Takara) in an SimpliAmp instrument (Applied Biosystems) with specific primer sets (Table 1). The PCR products were confirmed by electrophoresis on a 1.5% agarose gel containing ethidium bromide. The PCR conditions used were: 1 cycle at 98 °C for 30 s; 25–40 cycles at 98 °C for 10 s; 56–61 °C for 30 s; and 72 °C for 35 s; and 1 cycle at 72 °C for 2 min. The RT-PCR product sizes were 560 bp, A_1_AR; 502 bp, A_2A_AR; 506 bp, A_2B_AR; 558 bp, A_3_AR; 463 bp, CK14; 534 bp, TRPV4; 547 bp, VNUT; and 545 bp, GAPDH.

**Table 1:** Primer sequences for RT-PCR.

Primer name	Sequence (5′ → 3′)
A_1_A-F	CCCTGGCGGTAGCTGATGTGG
A_1_A-R	CAGCGACTTGGCGATCTTGAGC
A_2A_A-F	GCCTGCTTTGTCCTGGTCCTCAC
A_2A_A-R	GCAGGGGCAACCAGCAGAGG
A_2B_A-F	CGGTGGGAGCCTCGAGTGC
A_2B_A-R	CAGCATTATGAGCAGTGGAGGAAGGAC
A_3_A-F	GACAACACCACGGAGACGGACTG
A_3_A-R	GACGAGGATCCAGGTGACGAAGC
CK14-F	CAACAGCGAGCTGGTGCAGAG
CK14-R	GACCTGCTCGTGGGTGGAGAC
TRPV4-F	GGAGGAGAAAGGTCGTGGAGAAG
TRPV4-R	CGATGTGCGGCTGGTTGG
VNUT-F	GGCCCTGGGATTCCTTGCTCAAG
VNUT-R	CTCGATCAGGTAGCCACTCAGACACAC
GAPDH-F	TGAAGGGTGGAGCCAAAAGG
GAPDH-R	GGAAGAGTGGGAGTTGCTGTTG

### Immunostaining

For immunocytochemistry, esophageal keratinocytes of both strains were seeded as described above in 2-well culture inserts and left for 72 h to reach confluence. The cultured cells were fixed 24 h after insert removal with 4% paraformaldehyde for 10 min and washed in PBS. Cells were first incubated with blocking solution (PBS supplemented with 0.1% Triton X-100 and 3% BSA) for 20 min at room temperature and then incubated overnight at 4 °C with the primary antibody rabbit anti-CK14 (COVANCE) diluted 1:500 in blocking solution. The cells were washed (3 × 10 min) with PBS supplemented with 0.1% Triton X-100 and incubated with secondary antibody (Goat anti-rabbit IgG-Alexa488, 1:1,500; (Invitrogen, Inc.) for 1 h at room temperature. The cells were stained for 2 min with DAPI (1:1,000; Dojin Chemical Corp.) before a cover slip was applied. Images were acquired with a fluorescence microscope (BZ9000; Keyence, Osaka, Japan).

### Statistics

Data analysis was performed using SPSS version 23 (SPSS Inc., Chicago, IL, USA), and the results are expressed as means ± SEM with *n* representing the number of animals used. Statistical differences in the means between two groups were tested using unpaired (independent) Student t-test and paired samples t-test. One-way ANOVA test followed by a Tukey post-hoc test was used to compare the means of more than two groups. All *p* values are two-tailed and *p* < 0.05 was considered as significant for all statistical analyses in this study.

## Supplementary information

Supplementary file1 (DOCX 15 kb)

Supplementary file2 (AVI 39009 kb)

Supplementary file3 (AVI 38562 kb)

Supplementary file4 (JPG 295 kb)

Supplementary file5 (JPG 303 kb)

Supplementary file6 (JPG 547 kb)

Supplementary file7 (JPG 175 kb)

Supplementary file8 (JPG 310 kb)

Supplementary file9 (JPG 611 kb)

Supplementary file10 (JPG 129 kb)

## Data Availability

All data generated or analyzed during this study are included in this published article and its Supplementary Information File.

## Abbreviations

AR: Adenosine receptor
A_1_AR: A_1_ adenosine receptor
A_2A_AR: A_2A_ adenosine receptor
A_2B_AR: A_2B_ adenosine receptor
A_3_AR: A_3_ adenosine receptor
CK14: Cytokeratin 14
KO: Knockout
TRP: Transient receptor potential
TRPV4: Transient receptor potential vanilloid 4
VNUT: Vesicular nucleotide transporter
WT: Wild-type
