# Supplementary material for: Deletion of TRPV4 enhances in vitro wound healing of murine esophageal keratinocytes
Source: Sci Rep. 2020 Jul 9;10:11349. doi: 10.1038/s41598-020-68269-8 (PMC7347589; doi:10.1038/s41598-020-68269-8)
Supplement: Supplementary file 1 — Supplementary file1 (DOCX 15 kb) [file 41598_2020_68269_MOESM1_ESM.docx]

**Supplementary Figure S1.** Cytokeratin 14 (CK14) immunostaining of WT and TRPV4-KO esophageal keratinocytes 24 hours after insert removal.

**Supplementary Figure S2.** Representative fluorescence images of WT and TRPV4-KO esophageal keratinocytes transfected with DsRed-tagged mouse TRPV4 24 h after insert removal. Scale bar represents 200 μm.

**Supplementary Figure S3.** The effect of TRPV4 deletion on different cell cycle phases of esophageal keratinocytes. Representative images of cell cycle assay of WT and TRPV4-KO keratinocyte cultures at 0, 24, 48 and 72 h after insert removal. Pale yellow green for G0/G1 phase, green for S phase and dark blue for G2/M phase. Scale bar represents 200 μm.

**Supplementary Figure S4.** GAPDH, CK14 (keratinocyte marker), VNUT (vesicular nucleotide transporter) and TRPV4 mRNA transcription was examined with (+) and without (–) RT reaction. CK14, VNUT and GAPDH were transcribed in the mucosa of both strains, whereas TRPV4 was only transcribed in the WT mucosa.

**Supplementary Figure S5.** Phase-contrast microscopy images for WT and TRPV4-KO keratinocyte cultures 0 h after insert removal showing regular edges of cell-free gaps. The scale bar represents 200 μm.

**Supplementary Figure S6.** Quantification of gap area using ImageJ software. TRPV4-KO keratinocytes stained with calcein (1:1000) at (A) 24 and (B) 72 h after insert removal. The rectangular area is 0.5 mm x 2 mm (1 mm^2^). Scale bar represents 200 μm.

**Supplementary Figure S7.** Full-length gels showing adenosine receptor mRNA transcription with (+) and without (–) RT reaction. All adenosine receptor subtypes were transcribed in the esophageal mucosa of WT and TRPV4-KO mice, but A_2B_ adenosine receptor had apparently higher transcription compared to A_1_, A_2A_ and A_3_ adenosine receptors.

**Supplementary Video S1.** Time-lapse video showing esophageal keratinocyte *in vitro* wound healing in WT and TRPV4-KO mice. Video files were constructed from representative photos taken every 15 min for 72 h following insert removal. Scale bars represent 200 μm.
