# Supplementary figures and images for: Deletion of TRPV4 enhances in vitro wound healing of murine esophageal keratinocytes
Source: Sci Rep. 2020 Jul 9;10:11349. doi: 10.1038/s41598-020-68269-8 (PMC7347589; doi:10.1038/s41598-020-68269-8)

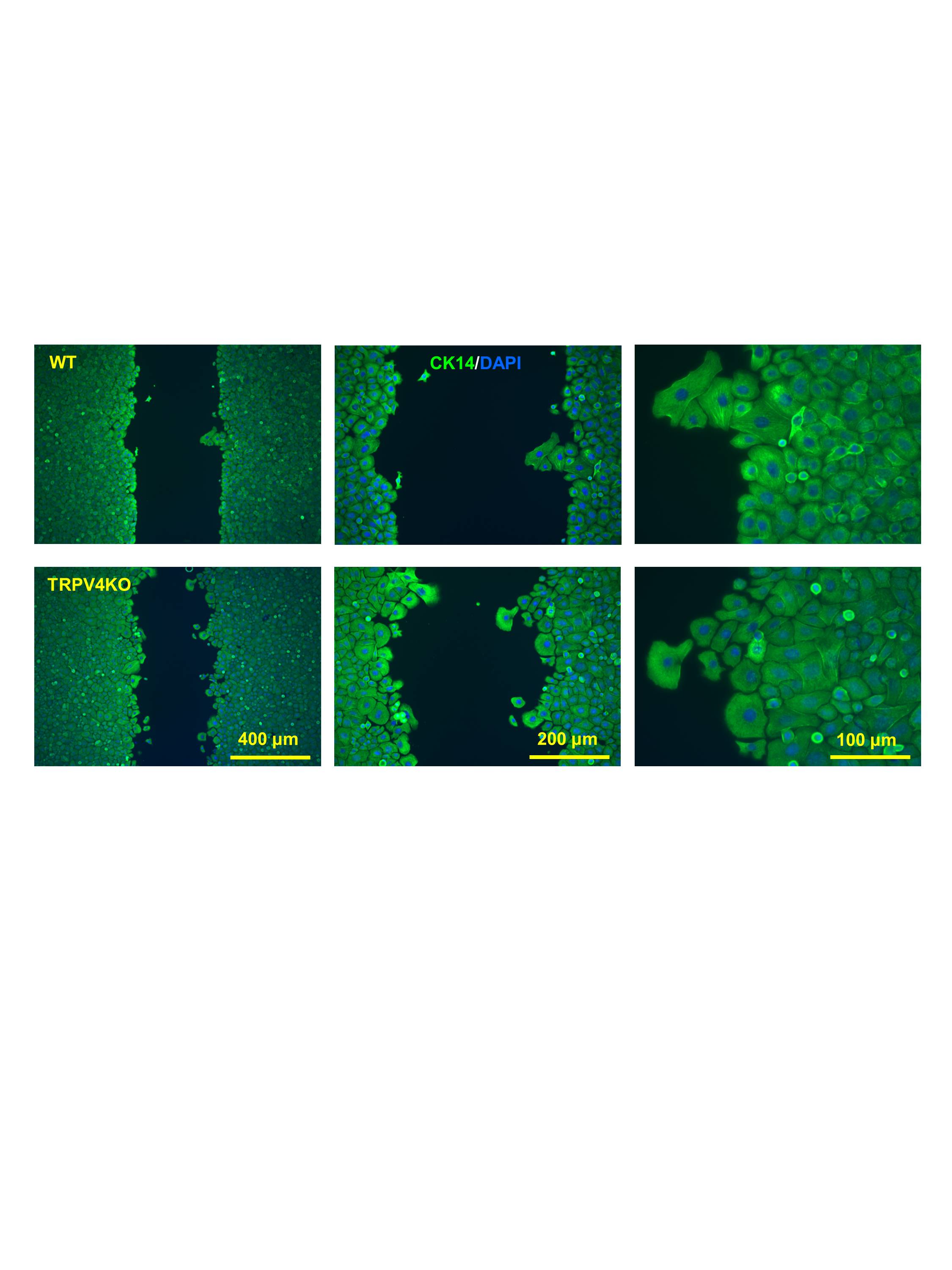

Supplement: Supplementary file 4 — Supplementary file4 (JPG 295 kb) [file 41598_2020_68269_MOESM4_ESM.jpg]

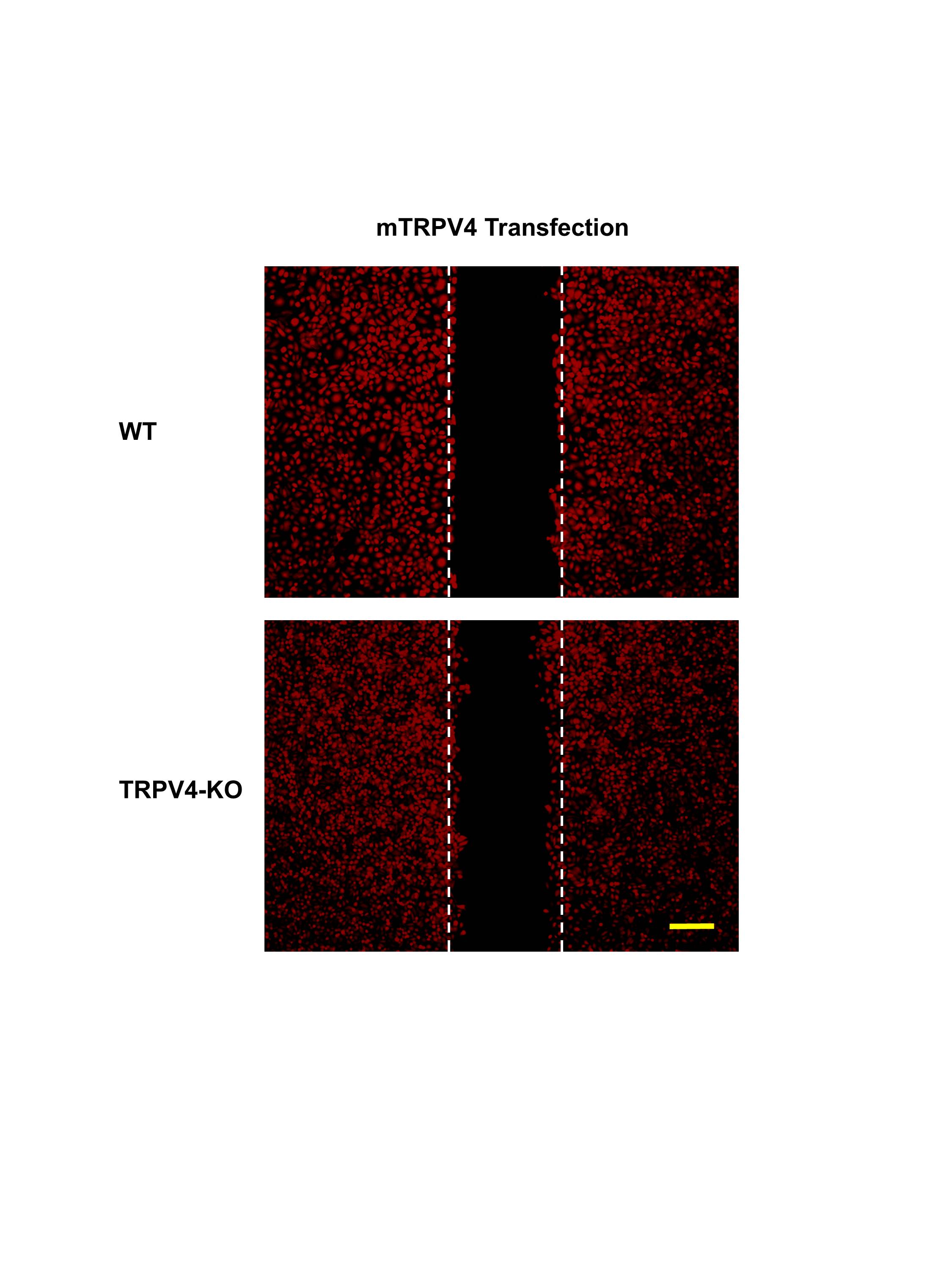

Supplement: Supplementary file 5 — Supplementary file5 (JPG 303 kb) [file 41598_2020_68269_MOESM5_ESM.jpg]

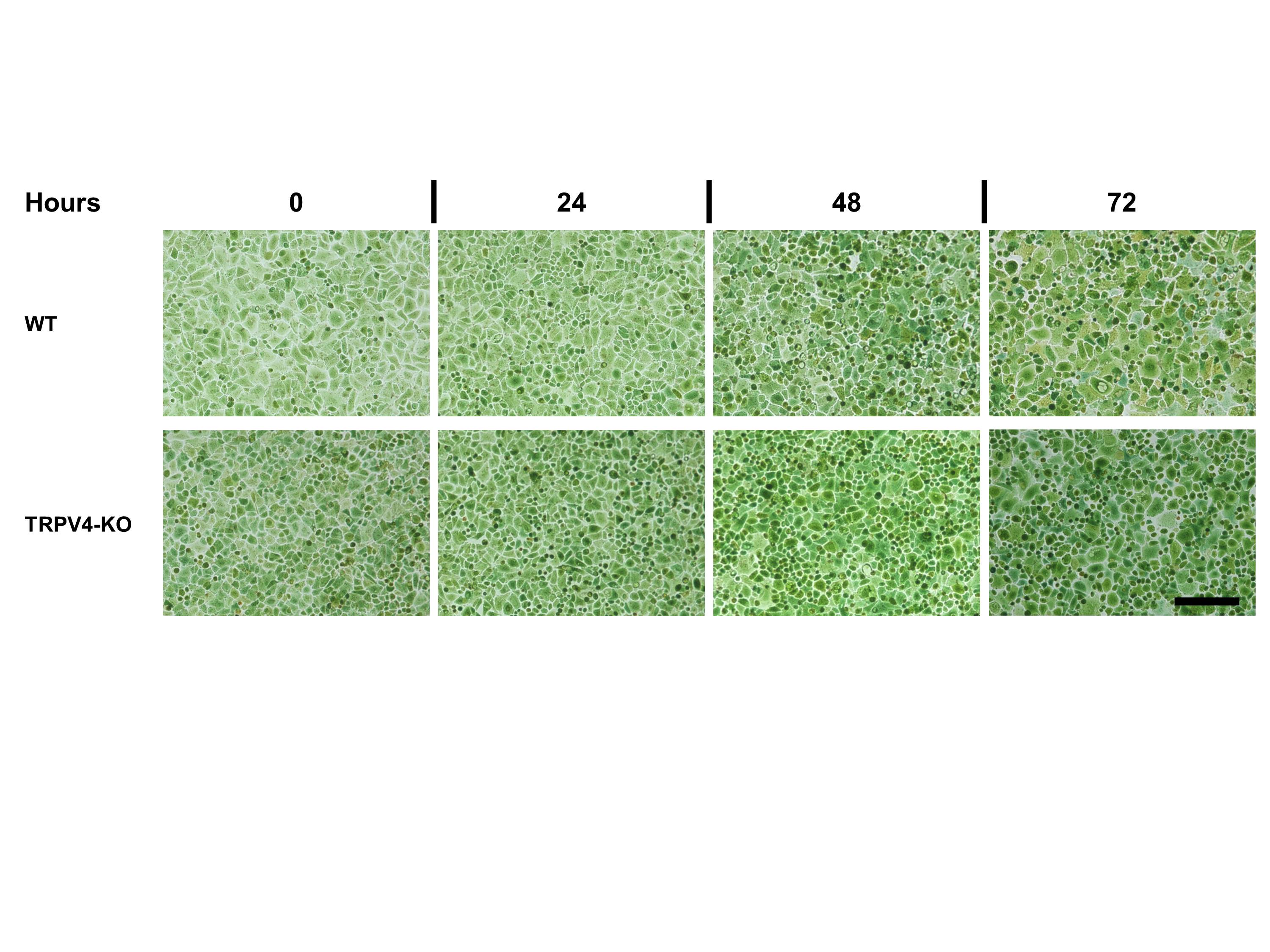

Supplement: Supplementary file 6 — Supplementary file6 (JPG 547 kb) [file 41598_2020_68269_MOESM6_ESM.jpg]

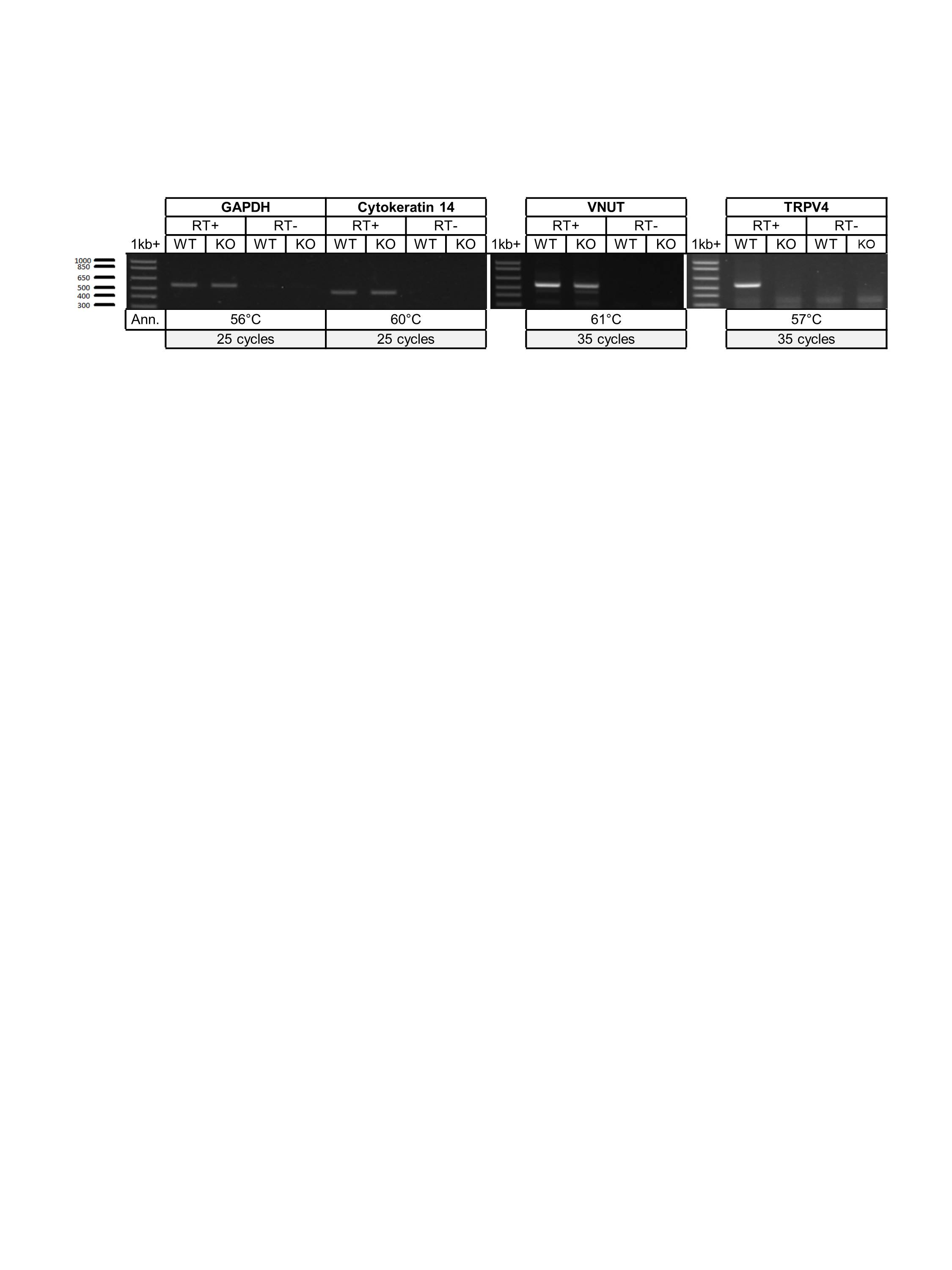

Supplement: Supplementary file 7 — Supplementary file7 (JPG 175 kb) [file 41598_2020_68269_MOESM7_ESM.jpg]

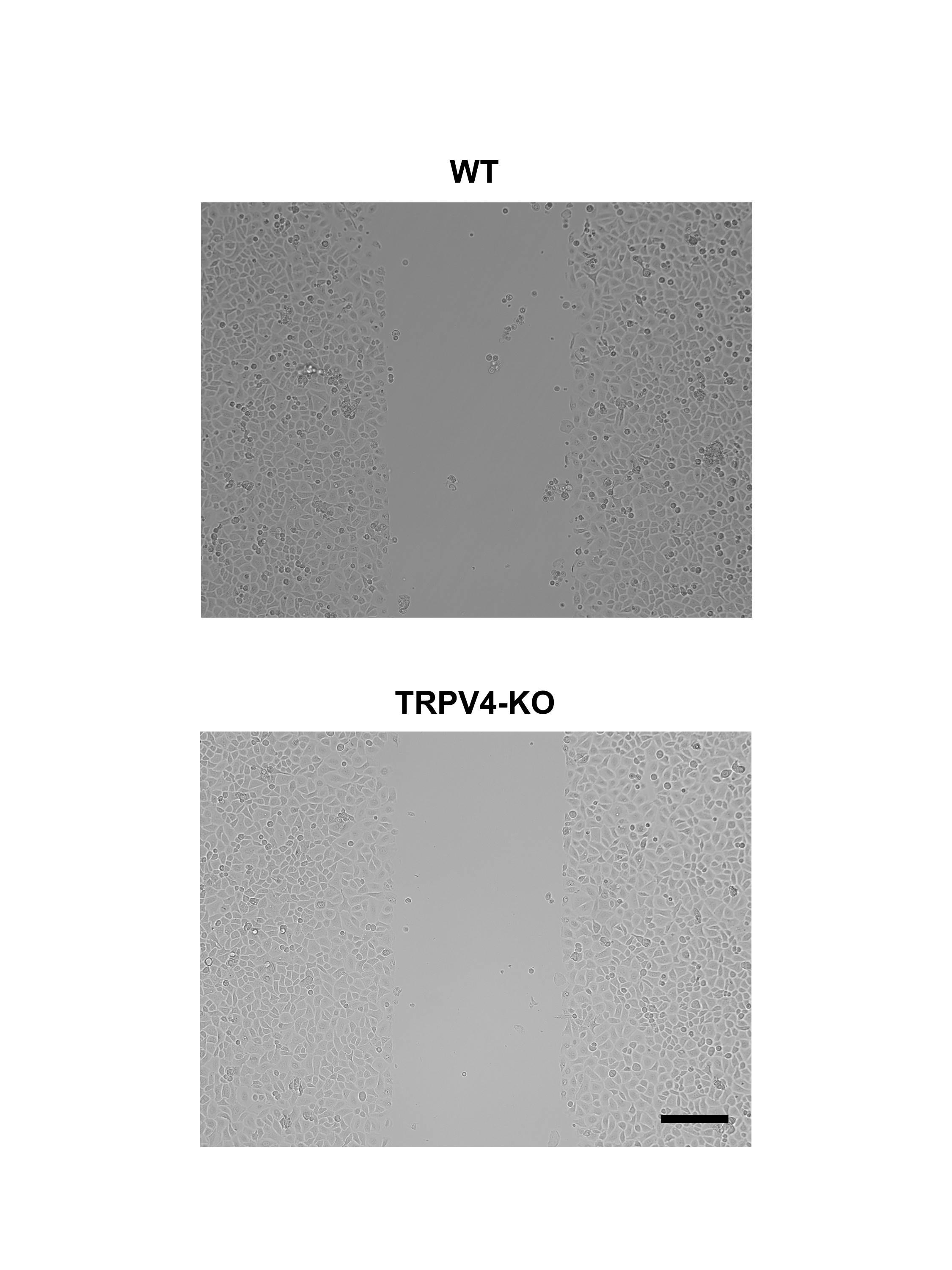

Supplement: Supplementary file 8 — Supplementary file8 (JPG 310 kb) [file 41598_2020_68269_MOESM8_ESM.jpg]

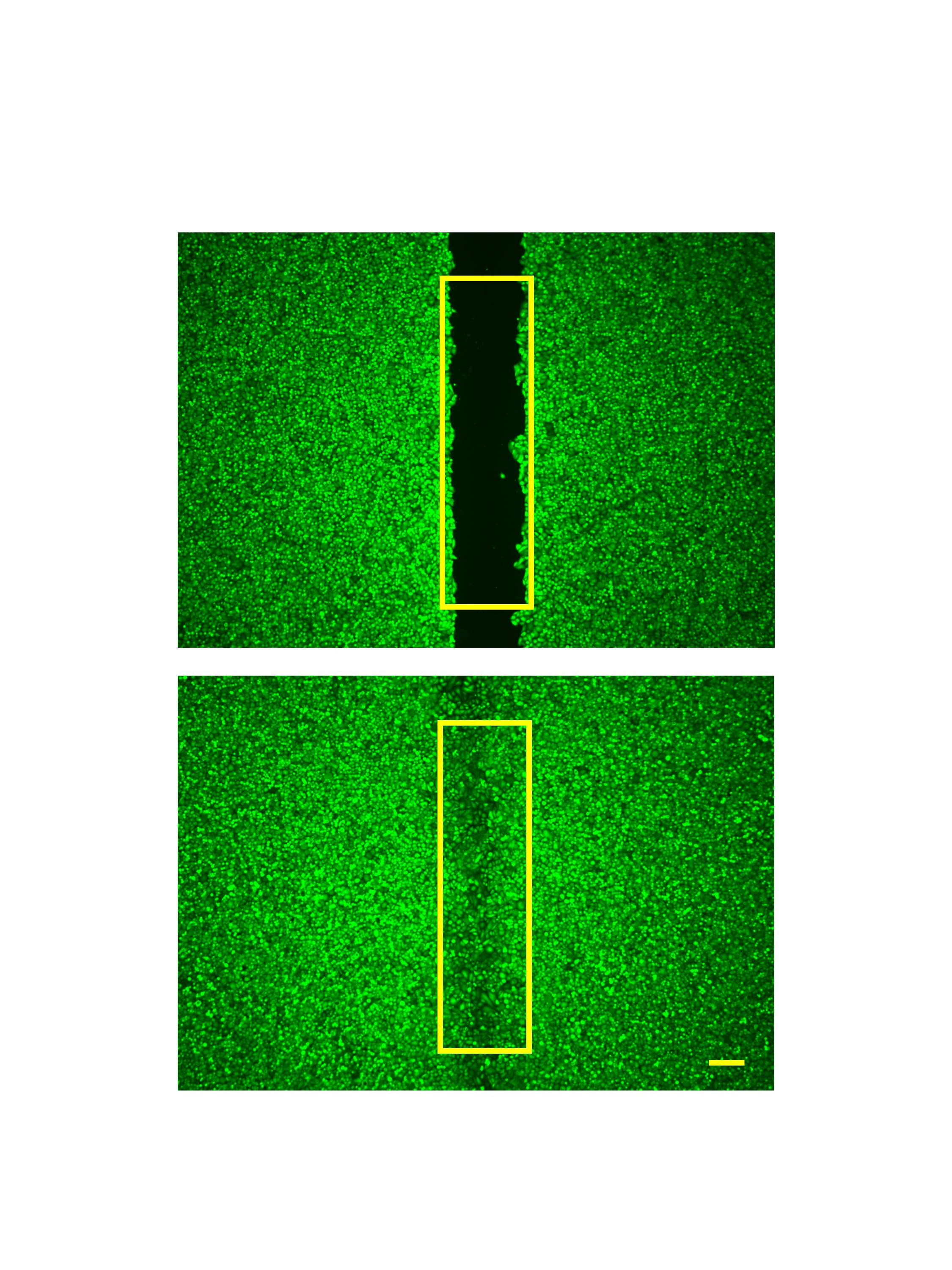

Supplement: Supplementary file 9 — Supplementary file9 (JPG 611 kb) [file 41598_2020_68269_MOESM9_ESM.jpg]

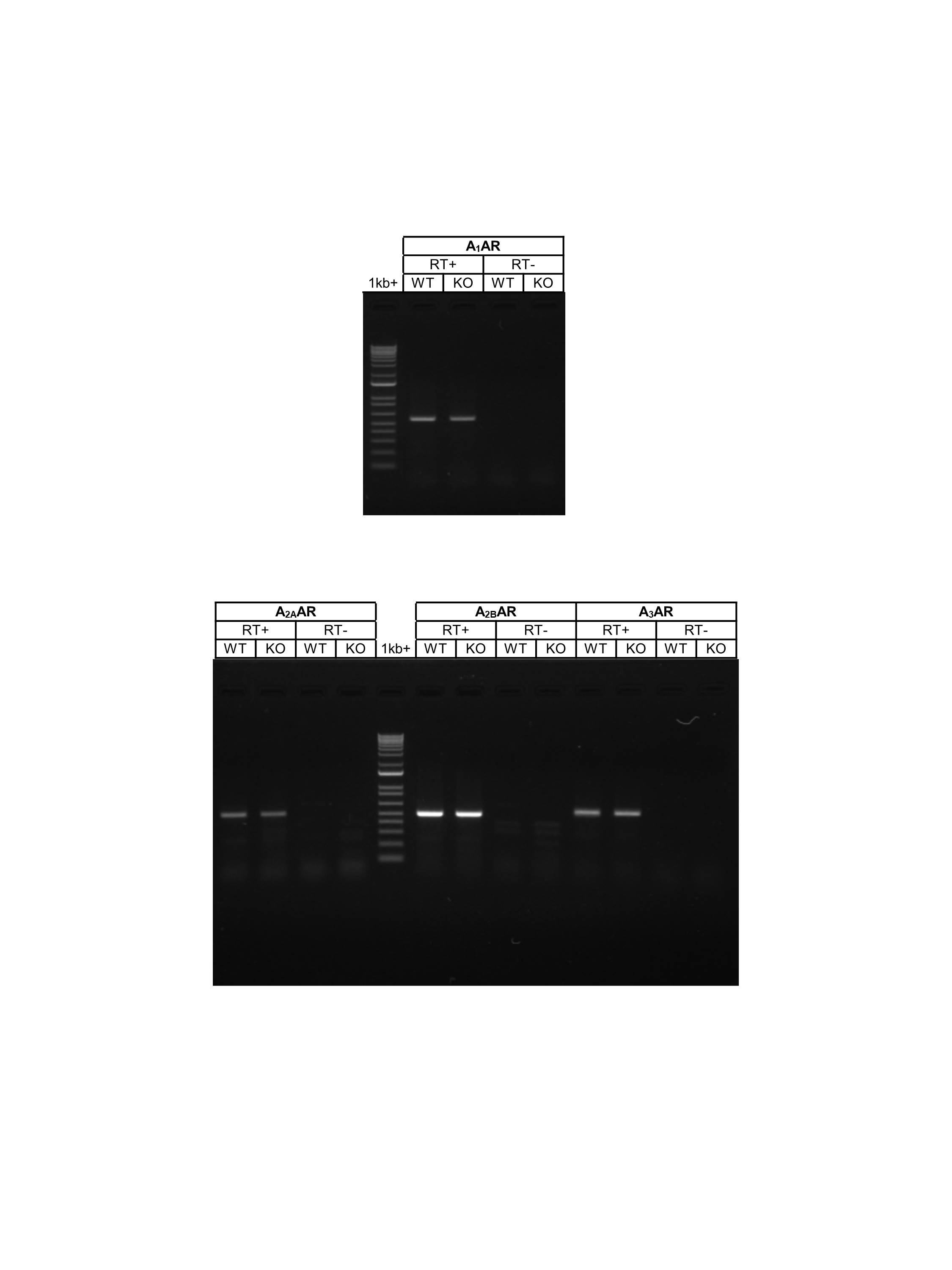

Supplement: Supplementary file 10 — Supplementary file10 (JPG 129 kb) [file 41598_2020_68269_MOESM10_ESM.jpg]
